# Big Data and Infectious Disease Epidemiology: Bibliometric Analysis and Research Agenda

**DOI:** 10.2196/42292

**Published:** 2023-03-31

**Authors:** Lateef Babatunde Amusa, Hossana Twinomurinzi, Edith Phalane, Refilwe Nancy Phaswana-Mafuya

**Affiliations:** 1 Centre for Applied Data Science University of Johannesburg Johannesburg South Africa; 2 Department of Statistics University of Ilorin Ilorin Nigeria; 3 Pan African Centre for Epidemics Research (PACER) Extramural Unit South African Medical Research Council/University of Johannesburg Johannesburg South Africa; 4 Department of Environmental Health Faculty of Health Sciences University of Johannesburg Johannesburg South Africa

**Keywords:** big data, bibliometrics, infectious disease, COVID-19, disease surveillance, disease, pandemic, data, surveillance, hotspot, epidemiology, social media, utility, electronic health records

## Abstract

**Background:**

Infectious diseases represent a major challenge for health systems worldwide. With the recent global pandemic of COVID-19, the need to research strategies to treat these health problems has become even more pressing. Although the literature on big data and data science in health has grown rapidly, few studies have synthesized these individual studies, and none has identified the utility of big data in infectious disease surveillance and modeling.

**Objective:**

The aim of this study was to synthesize research and identify hotspots of big data in infectious disease epidemiology.

**Methods:**

Bibliometric data from 3054 documents that satisfied the inclusion criteria retrieved from the Web of Science database over 22 years (2000-2022) were analyzed and reviewed. The search retrieval occurred on October 17, 2022. Bibliometric analysis was performed to illustrate the relationships between research constituents, topics, and key terms in the retrieved documents.

**Results:**

The bibliometric analysis revealed internet searches and social media as the most utilized big data sources for infectious disease surveillance or modeling. The analysis also placed US and Chinese institutions as leaders in this research area. Disease monitoring and surveillance, utility of electronic health (or medical) records, methodology framework for infodemiology tools, and machine/deep learning were identified as the core research themes.

**Conclusions:**

Proposals for future studies are made based on these findings. This study will provide health care informatics scholars with a comprehensive understanding of big data research in infectious disease epidemiology.

## Introduction

Globally, the infectious disease burden continues to be substantial in countries with low and lower-middle income, while morbidity and mortality related to neglected tropical diseases and HIV infection, tuberculosis, and malaria remain high. Tuberculosis and malaria are endemic to many areas, imposing substantial but steady burdens. At the same time, other infections such as influenza fluctuate in pervasiveness and intensity, disrupting the developing and developed settings alike when an outbreak and epidemic occurs. Additionally, deaths have persisted over the 21st century due to emerging and reemerging infectious diseases compared with seasonal and endemic infections. This portrays a new era of infectious disease, defined by outbreaks of emerging, reemerging, and endemic pathogens that spread quickly with the help of global mobility and climate change [1].

Moreover, the risk from infectious diseases is globally shared. While infectious diseases thrive in underresourced settings, inequalities and inequities in accessing health and health care create a favorable environment for infectious diseases to spread [2,3]. Addressing inequalities and inequities in accessing health care, and improving surveillance and monitoring of infectious diseases should be prioritized to minimize the emergence and spread of infections.

Recent years have witnessed the rapid emergence of big data and data science research, propelled by the increasing availability of digital traces [4]. The growing availability of electronic records and passive data generated by social media, the internet, and other digital sources can be mined for pattern discoveries and knowledge extraction. Like most buzz words, *big data* has no straightforward meaning and its definition is evolving. Broadly, big data refers to a large volume of structured or unstructured data, with *largeness* itself associated with three major terms known as the “3 Vs”: volume (large quantity), velocity (coming in at unprecedented real-time speeds), and variety (increasing collection from different data sources). Additional characteristics of big data include veracity, validity, volatility, and value [5]. For epidemiology and infectious diseases research, this means that in the last decade, there has been a significant spike in the number of studies with considerable interest in using digital epidemiology and big data tools to enhance health systems in terms of disease surveillance, modeling, and evidence-based responses [4,6-8]. Digital epidemiology uses digital data or online sources to gain insight into disease dynamics and health equity, and to inform public health programs and policies [9,10].

The success of infectious disease control relies heavily on surveillance systems tracking diseases, pathogens, and clinical outcomes [11]. However, conventional surveillance systems are known to frequently have severe time lags and limited spatial resolution; therefore, surveillance systems that are robust, local, and timely are critically needed. It is crucial to monitor and forecast emerging and reemerging infections [12] such as severe acute respiratory syndrome, pandemic influenza, Ebola, Zika, and drug-resistant pathogens, especially in resource-limited settings such as low-middle–income countries. Using big data to strengthen surveillance systems is critical for future pandemic preparedness. This approach provides big data streams that can be triangulated with spatial and temporal data. These big data streams include digital data sources such as mobile health apps, electronic health (or medical) records, social media, internet searches, mobile phone network data, and GPS mobile data. Many studies have demonstrated the usefulness of real-time data in health assessments [13-18]. Some of these studies have been used explicitly for the monitoring and forecasting of epidemics such as COVID-19 [19], Zika [13], Ebola [16], and influenza [14].

The body of extant literature at the nexus of big data, epidemiology, and infectious diseases is rapidly growing. However, despite its growth and dispersion, there has been a limited synthesis of the applications. A previous study [20] performed a bibliometric analysis focusing on only HIV. A bibliometric analysis is a statistical or quantitative analysis of large-scale bibliographic metadata (or metrics of published studies) on a given topic. These quantitative analyses detect patterns, networks, and trends among the bibliographic metadata [21,22]. Thus, the aim of this study was to address the evolution of big data in epidemiology and infectious diseases to identify gaps and opportunities for further research. The study findings reveal interesting patterns and can inform trending research focus and future directions in big data–driven infectious diseases research.

## Methods

### Study Design

A bibliometric analysis was performed to understand and explore research on big data in infectious disease modeling and surveillance. The adopted bibliometric methodology involved three main phases: data collection, data analysis, and data visualization and reporting [23].

### Search Strategy

Regarding data collection, which entails querying and exporting data from selected databases, we queried the Web of Science (WoS) core databases for publications using specific inclusion and exclusion criteria. Compared to other databases, the WoS has been shown to have better quality bibliometric information [23,24] and more excellent coverage of high-impact journals [25]. With the aid of domain knowledge experts from the fields of both big data and epidemiology, we iteratively developed a search strategy and selected the following search terms. The following search string queried all documents’ titles, abstracts, and keywords, and generated 3235 publications in the WoS collection:

(Epidemic* OR “infectious disease*” OR “Disease surveillance” OR “disease transmission” OR “disease outbreak*” OR (“communicable disease*” NOT “non-communicable disease”) OR syndemic* OR HIV OR AIDS OR “human immunodeficiency virus” OR coronavirus* OR SARS-CoV-2 OR COVID-19 OR Influenza OR flu OR Zika OR Ebola OR MERS OR “Middle East respiratory syndrome” OR Tuberculosis OR “Monkey Pox” OR “Dengue virus” OR Hepatitis*)

*AND*

(“BIG DATA” OR “web mining” OR “opinion mining” OR “Google Trend*” OR “Google search*” OR “Google quer*” OR “Internet search*” OR “Internet quer*” OR “search engine quer*” OR “Digital traces” OR “electronic health records” OR “Digital epidemiology”)

### Screening Strategy

**Figure 1:**
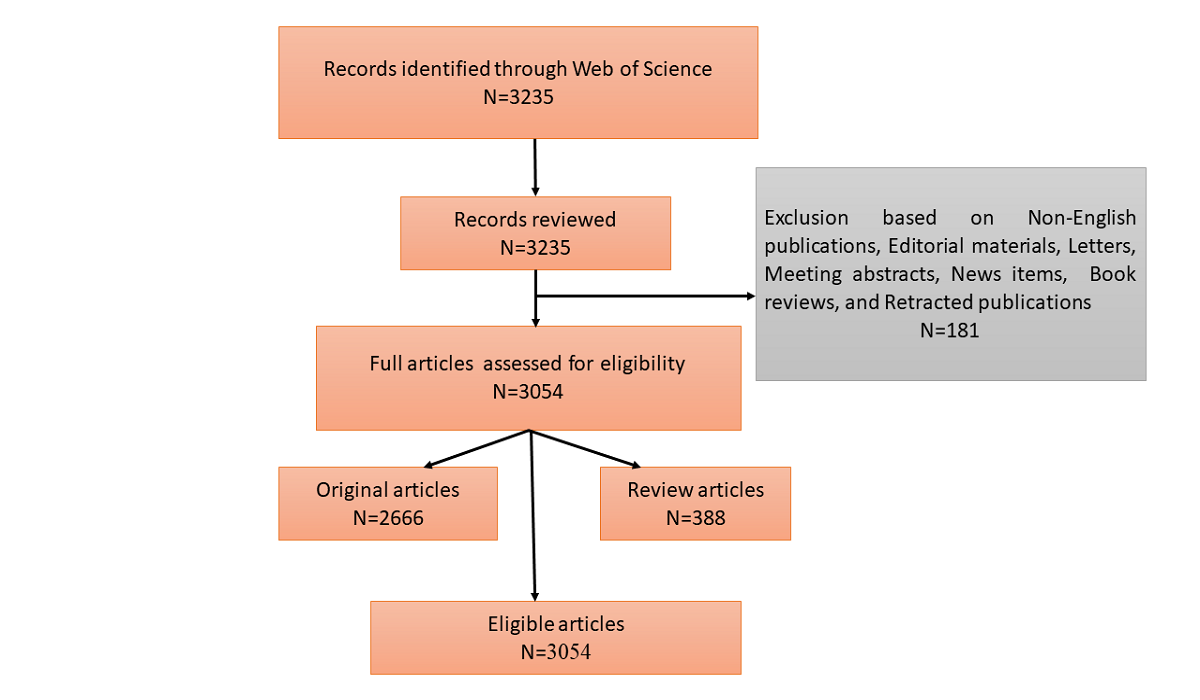
Flow chart of the literature selection process.

Documents not written in English and not peer-reviewed, including editorial materials, letters, meeting abstracts, news items, book reviews, and retracted publications, were removed from the data set given the focus on bibliometric analysis, leaving 3054 documents for the analytic sample (Figure 1).

### Analysis

The 3054 bibliographic data were exported into the R package *bibliometrix* [23] for analysis. This package was specifically used to conduct performance analysis and science mapping of big data in infectious disease epidemiology. Performance mapping evaluates the production and impact of research constituents, including authors, institutions, countries, and journals. Science mapping examines the relationships between the research constituents by analyzing the topic’s conceptual, intellectual, and social structure.

There are several metrics available for bibliometric analysis. In this study, the primary metrics used for evaluating productivity and influence were the H-index and M-index. The H-index represents the number of published papers *h*, such that the citation number is at least *h* [26]. The H-index can be computed for different bibliometric units of analysis: authors, journals, institutions, and countries. The M-index simply adjusts the H-index for the academic age (ie, the number of years since the researcher’s first publication). Other utilized performance analysis metrics were obtained from yearly research output and citation counts. These metrics also contribute to identifying the main themes and the key actors in the research area.

In terms of science mapping, network maps were constructed for some selected bibliographic units of analysis [27]. These networks exhibit frequency distributions of the involved bibliographic data over time. For instance, international collaborations can be explored by assessing same-country publications. A cocitation network analysis was also used to analyze publication references. In addition, using the Louvain clustering algorithm and a greedy optimization technique [28], a co-occurrence analysis was used to understand the conceptual structure of the research area. The basic purpose of co-occurrence analysis is to investigate the link between keywords based on the number of times they appear together in a publication. Notable research topics and over-time trends were detected by generating clusters for author-provided keywords [29]. VOSviewer [30] was used to construct the network visualizations. Each network node represents a research constituent (eg, author, country, institution, article, document source, keyword). The node’s size is proportional to the occurrence frequency of the relevant parameters. The degree of association is represented by the thickness of the link between nodes, and the various colors reflect distinct clusters.

## Results

### Descriptive Summary

**Figure 2:**
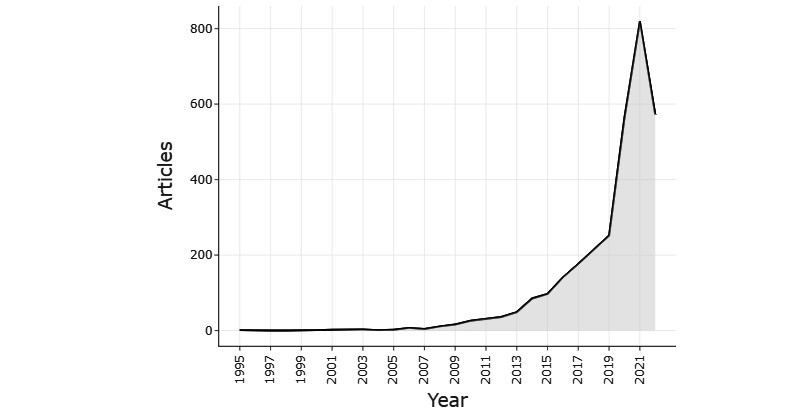
Annual growth of publications related to big data in infectious diseases research.

The bibliographic data set comprises 3054 documents from 1600 sources, 14,351 authors, and 121,726 references. From the 3054 documents, 2666 (87.30%) were original research articles and the remaining 388 (12.70%) were review papers. The research output before 2009 was relatively low. The annual publication output during the 27 years (1995-2022) grew steadily, with a yearly growth rate of 26.5%. The publication growth increased steeply between 2013 and 2020 (Figure 2). Table 1 presents the summary statistics of the primary characteristics of these 3054 publications, including the time span and information about documents and authors.

As shown in Table 2, the most productive and influential sources publishing on topics related to big data and infectious diseases epidemiology were *Journal of Medical Internet Research* and *PLoS One* (H-index=18), followed by *IEEE Access* (H-index=13). In terms of productivity, *Journal of Medical Internet Research* produced a slightly higher number of publications (n=61) than the next best journal *PLoS One* (n=56). *PLoS One* had the highest number of total citations at 1893.

As shown in Table 3, the most productive and influential author was Zhang Y (H-index=17), followed by Li X (H-index=13) and Wang J (H-index=12). Wang L had the highest total citations (n=1072), which was substantially higher than the next most impactful author Wang J (total citations=861).

**Table 1:** Main descriptive summary of the extracted bibliographic records from 1995 to 2022.

Description		Results
Time span (years)		1995-2022
Sources, n		1600
Documents, n		3054
Annual growth rate, %		26.52
Document average age (years)		2.86
Average citations per document, n		18.52
References, n		121,726
**Authors, n**		
	Total	14,351
	Single-authored documents	225
**Author collaborations**		
	Single-authored documents, n	236
	Coauthors per document, n	5.55
	International coauthorships, %	28.04

**Table 2:** Top 10 productive and influential publication sources ranked by H-index.

Journal	Aim and scope	H-index	M-index	Total citations, n	Publications, n	Publication year
Journal of Medical Internet Research	Digital health, data science, health informatics, and emerging technologies for health, medicine, and biomedical research	18	1.13	1705	61	2007
PLoS One	Multidisciplinary	18	1.39	1893	56	2010
IEEE Access	Multidisciplinary, comprising all IEEE fields of interest, emphasizing applications-oriented and interdisciplinary articles	13	1.63	983	32	2015
Scientific Reports	Publishes from all areas of the natural sciences, psychology, medicine, and engineering	13	1.63	389	23	2015
Journal of the American Medical Informatics Association	Biomedical and health informatics, including clinical care, clinical research, translational science, implementation science, imaging, education, consumer health, public health, and policy	12	0.86	569	33	2009
BMJ Open	Medical journal considering papers in clinical medicine, public health, and epidemiology	11	1.10	310	32	2013
JMIR Public Health & Surveillance	Multidisciplinary journal with a unique focus on the intersection of innovation and technology in public health	11	2.20	724	23	2018
International Journal of Medical Informatics	Medical informatics, including information systems and computer-aided medical support decision systems	11	0.65	450	16	2006
International Journal of Infectious Diseases	Original clinical and laboratory-based research, together with reports of clinical trials, reviews, and some case reports dealing with the epidemiology, clinical diagnosis, treatment, and control of infectious diseases	10	0.91	530	17	2012
BMC Medical Informatics & Decision Making	Relating to the design, development, implementation, and evaluation of health information technologies and decision-making for human health	10	0.91	208	15	2012

**Table 3:** Top 10 productive and influential authors ranked by H-index and total citations.

Author	H-index	M-index	Total citations, n	Publications, n	Publication year
Zhang Y	17	—^a^	776	35	—
Li X	13	—	544	35	—
Wang J	12	1.33	861	24	2014
Wang L	12	—	1072	22	—
Wang Y	10	1.25	342	21	2015
Li Z	10	1.67	366	14	2017
Brownstein JS	10	0.77	748	11	2010
Wang Z	9	1.00	427	18	2014
Zhang W	9	1.13	556	12	2015
Zhang X	9	1.29	371	12	2016

^a^Not available.

The aim and scope of the top 10 most influential journals, as listed in Table 2, is to publish medical research, medical informatics, or multidisciplinary studies. It can thus be inferred that major future breakthroughs regarding big data in infectious diseases epidemiology will likely appear in these journals.

**Figure 3:**
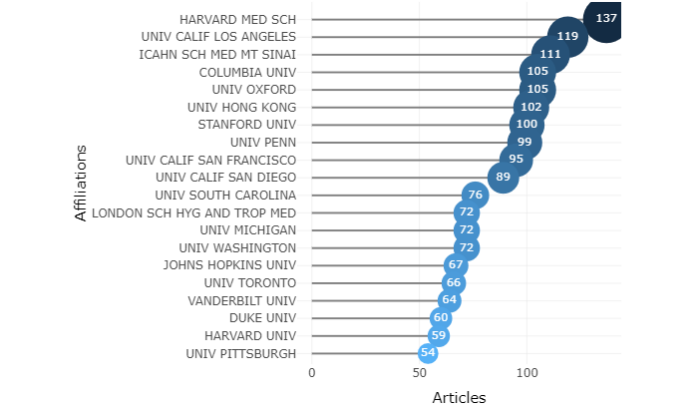
Top 20 institutions by number of publications. CALIF: California; HARVARD MED SCH: Harvard Medical School; ICAHN SCH MED MT SINAI: Icahn School of Medicine at Mount Sinai; LONDON SCH HYG AND TROP MED: London School of Hygiene & Tropical Medicine; PENN: Pennsylvania; UNIV: University.

Figure 3 displays the top 20 most productive institutions. Institutional contributions were assessed by affiliations with at least one author in the publication. Except for the University of California, the top three institutions, which account for 21.3% of the number of publications in the top 20, were medical schools: Harvard Medical School (7.9%) and Icahn School of Medicine at Mount Sinai (6.4%). The other institutions, each accounting for more than 6% of the total, included Columbia University and Oxford University in the top 5, whereas others in the top 20 are research universities: London School of Hygiene and Tropical Medicine focuses on global and public health, Taipei Medical University is medical-based, and Huazhong University of Science and Technology is focused on science and technology. The United States produced the majority of the top 10 most productive institutions, which were in the top 5.

**Figure 4:**
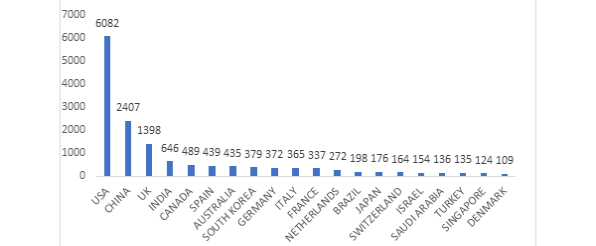
Top 20 productive countries by number of publications.

The 20 most productive countries (Figure 4) are led by the United States and China, accounting for more than half (57.3%) of the total publication output. The United States alone accounted for 41.1% of the productivity in this field. The other countries in the top five were the United Kingdom (9.4%), India (4.4%), and Canada (3.3%).

**Figure 5:**
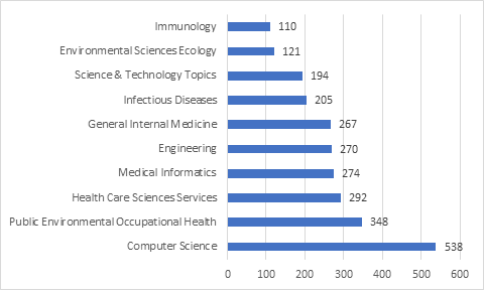
Top 10 key subject areas by number of publications.

Computer science was the most productive research domain in the bibliographic collection (Figure 5), accounting for 17.6% of the top 10 subject areas. In order of productivity, the other research subjects in the top 5 were public environmental and occupational health (11.4%), health care services (9.6%), medical informatics (9.0%), and engineering (8.8%).

**Figure 6:**
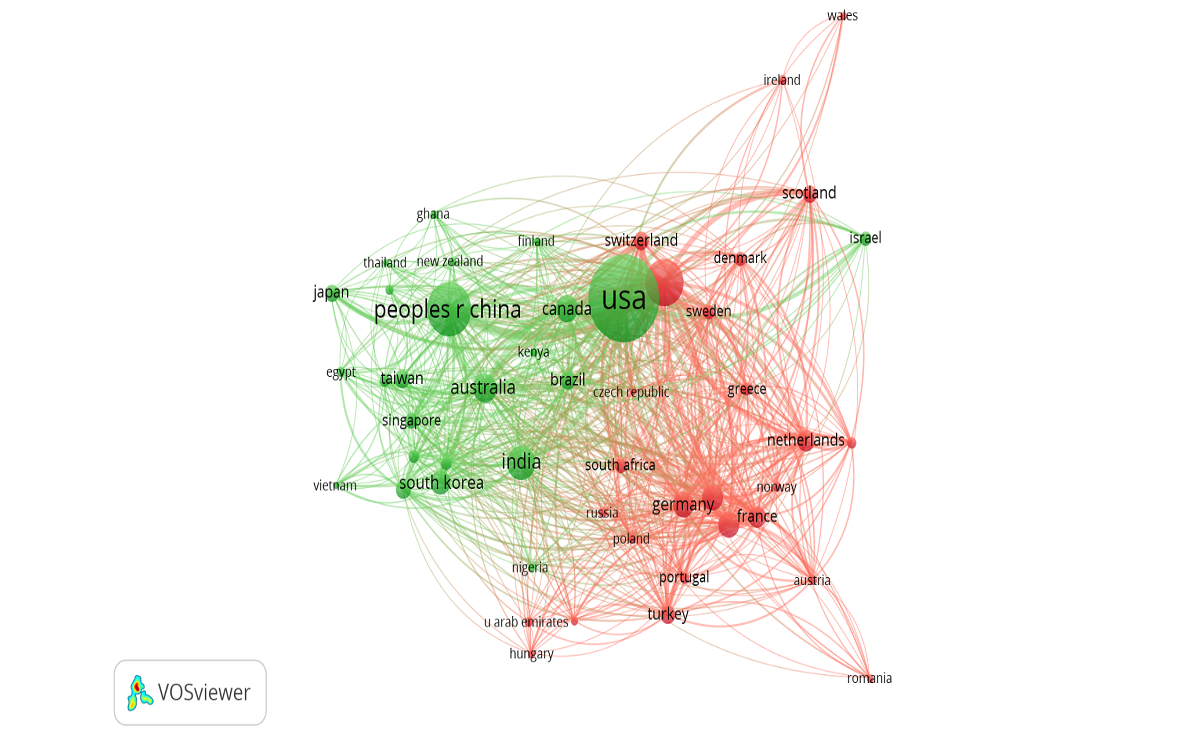
Network of country collaborations (≥10 documents, 50 countries, 2 clusters).

Two major clusters of countries represent the collaboration patterns of the most productive countries (Figure 6). The network was set to include only countries with at least 10 documents, resulting in 50 productive countries. The clustering results demonstrated a demarcation of European countries from the others. For instance, cluster 1 (red) represented most countries from Europe, with England, Germany, and Spain being the core countries. Non-European countries constituted the second cluster (green). The United States and China were the core countries of this group.

Regarding collaboration strength, the United States, with a total link strength of 570, featured the highest number of partners (48), accounting for almost all 50 countries in the network (96%). China, which distantly followed the United States, featured 38 partners and a total link strength of 304. This implies that collaboration is mainly regional.

**Figure 7:**
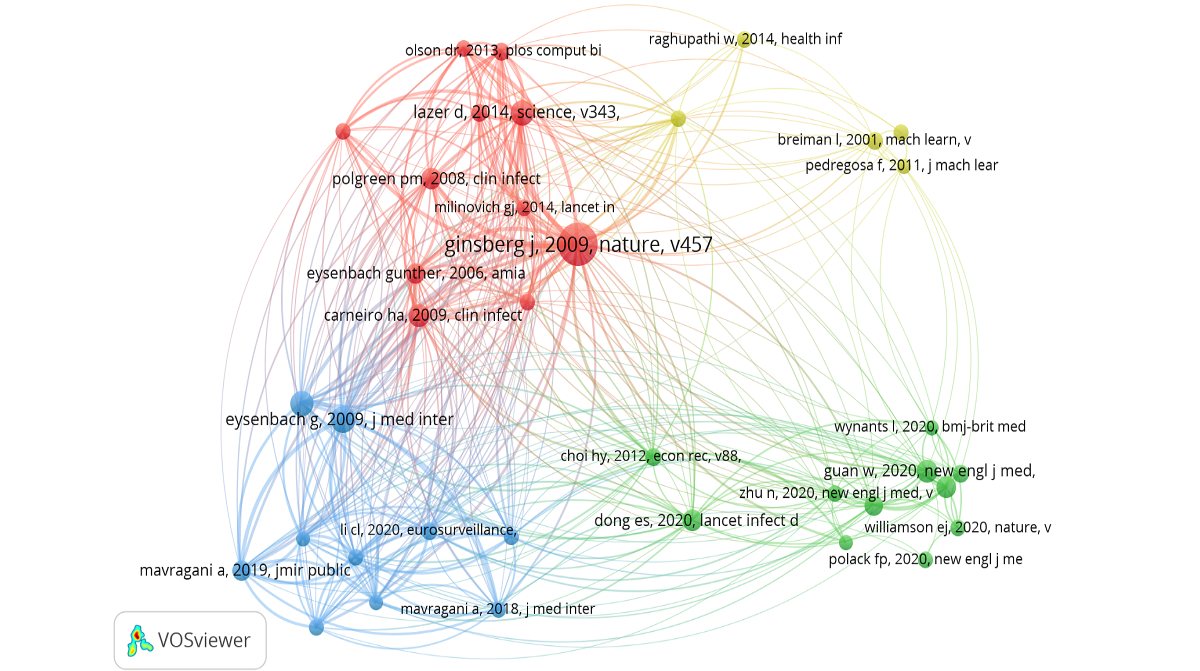
Network of cocited references.

Figure 7 shows a network map of cocited references in this research area, wherein the node’s size represents the citation strength of the individual studies. The network was set to include only studies with at least 25 citations, resulting in 37 studies. Ginsberg et al [31] published the most highly cited article (185 citations). This 13-year-old study presented a method that used Google search queries to track flu-like illnesses in a population. The second most cited study by Eysenbach [9] introduced the concept of infodemiology, the science of using the internet (eg, social media, search engines, blogs, and websites) to inform public health and public policy. Table 4 further summarizes the top 15 most cited references, including the title, year of publication, number of citations, type of disease, and data source.

The 37 studies in the network map of cocited references produced four thematic clusters (Figure 7); disease monitoring and surveillance (cluster 1), utility of electronic health (or medical) records (cluster 2), methodology framework for infodemiology tools (cluster 3), and machine learning and deep learning methods (cluster 4) were the main topics discussed.

**Figure 8:**
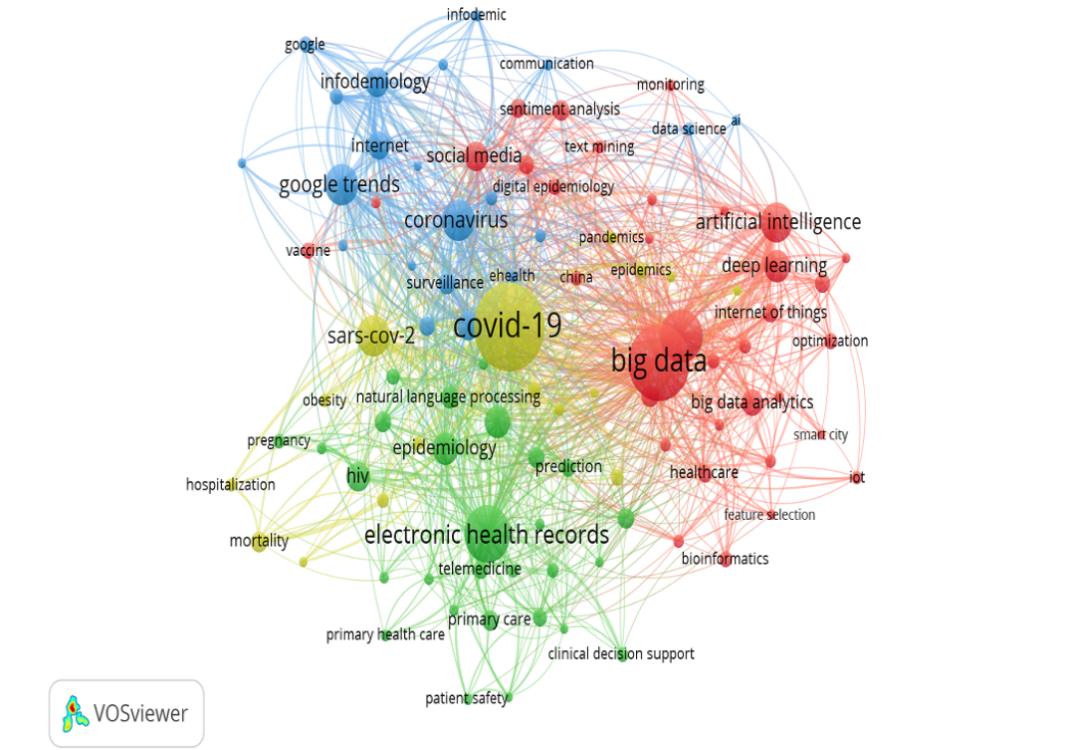
Co-occurrence networks of author keywords.

Keyword co-occurrence analysis serves as a supplement to enrich the understanding of the thematic clusters derived from the reference cocitation analysis and helps identify the core topics and contents [29]. As shown in Figure 8, the co-occurrence network displayed 100 relevant keywords after assigning a selection threshold of 10 for the number of keyword occurrences. The top 5 most frequently used keywords were *COVID-19, big data, machine learning, coronavirus,* and *electronic health records.*

**Table 4:** Summary of the top 15 most cited references.

Reference	Citations, n	Disease	Data source
Ginsberg et al [31]	185	Influenza	Google Trends
Eysenbach [9]	74	Influenza	NA^a^
Nuti et al [32]	69	NA	Google Trends
Lazer et al [33]	67	Influenza	Google Flu
Carneiro and Mylonaki [34]	54	NA	Google Trends
Zhou et al [35]	49	COVID-19	Electronic health records
Dong et al [36]	49	COVID-19	Twitter feeds and DXY^b^
Polgreen et al [37]	48	Influenza	Yahoo searches
Mavragani and Ochoa [38]	43	NA	Google Trends
Huang et al [39]	42	COVID-19	Electronic medical records
Eysenbach [40]	41	Influenza	Google Trends
Wu et al [41]	34	COVID-19	Electronic medical records
Li et al [42]	33	COVID-19	Internet searches^c^ and Weibo index^d^
Santillana et al [43]	31	Influenza	Twitter and Google Trends
Signorini et al [44]	30	Influenza	Twitter

^a^NA: not applicable (eg, a review paper, no particular disease or data source for a case study).

^b^Online platform of real-time COVID-19 cases in China.

^c^Internet searches include Google Trends and Baidu Index.

^d^Weibo is a China-based social media platform.

The 100 author-derived keywords produced four clusters from the coword analysis (Figure 8). Cluster 1 (yellow-green) is related to public health and infectious diseases, with top keywords such as *COVID-19, SARS-CoV-2, epidemiology,* and *epidemics*. Cluster 2 (green) is related to electronic storage and delivery of health care, with top keywords including *electronic health records, clinical decision support, primary care, epidemiology,* and *telemedicine*. Cluster 3 (blue) involves infodemiology tools, with top keywords including *coronavirus, google trends, social media, infodemiology*, and *surveillance*. Cluster 4 (red) is more coherent and broadly related to big data and artificial intelligence, including top keywords *big data, machine learning, artificial intelligence, deep learning,* and *big data analytics.*

### Systematic Review of the Top 20 Papers

Further filtering of the top 20 papers was performed to determine if they met the following criteria: (1) addressed at least one infectious disease and (2) utilized a big data source. A review of these 20 papers (summarized in Table 5) was then performed. These selected studies were mainly characterized by papers that utilized novel data sources, including internet search engine data (Google Trends: n=11; Baidu or Weibo index: n=2; Yahoo: n=1) and social media data (Twitter: n=5). Other data sources included electronic health or medical records (n=3) and Tencent migration data (n=1). The most frequently studied diseases were COVID-19 (n=10) [35,36,39,42,45-50], followed by influenza (n=8) [37,40,43,44,51-54]. Only one study considered the Zika virus [55], and another considered the trio of meningitis, legionella pneumonia, and Ebola [56].

**Table 5:** Summary of top 20 studies that addressed an infectious disease and utilized a big data source.

Rank	Study	Research objective and key findings	Infectious disease(s)	Big data source
1	Polgreen et al [37]	Used internet search engine data for infectious diseases epidemiology and examined the relationship between Yahoo search queries for influenza and actual influenza occurrence. They estimated linear models, using searches with 1-10–week lead times as explanatory variables to predict the percentage of cultures positive for flu and deaths attributable to pneumonia and influenza in the United States. The fitted models predicted an increase in cultures positive for influenza 1-3 weeks in advance of when they occurred (*P*<.001), and similar models predicted an increase in mortality attributable to pneumonia and influenza up to 5 weeks in advance (*P*<.001).	Influenza	Internet search engine
2	Walker et al [45]	The research explored internet activity related to loss of smell in the United States and seven European countries. Spearman rank correlation was used to assess the relationship between loss-of-smell relative search volumes (RSVs), with the daily confirmed cases of COVID-19 and deaths. Strong and significant correlations (*P*<.05) between daily RSVs related to loss of smell, daily COVID-19 cases, and deaths were found, ranging from 0.633 to 0.952.	COVID-19	Google Trends
3	Effenberger et al [46]	Studied correlations between RSVs and the official COVID-19 cases reported by the European Centre for Disease Control (ECDC) for some selected countries. They opted for time-lag correlation analysis and observed a time lag of –11.5 days being the highest correlation across all investigated countries.	COVID-19	Google Trends
4	Ayyoubzadeh et al [47]	Opted for machine/deep learning with Google Trends data. Linear regression and long short-term memory (LSTM) models were used to estimate COVID-19 cases. They found that the linear regression model had the smaller root mean square error (RMSE) and was the better predictive model. They also found the most predictive factors of the model to be search terms of handwashing, hand sanitizer, and antiseptic topics.	COVID-19	Google Trends
5	Husnayain et al [48]	Considered smaller spatial coverages in their Google Trends analysis. They retrieved data from specific locations and subregions in Taiwan nationwide using defined search terms related to the coronavirus, handwashing, and face masks. Their findings suggest high to moderate correlations between RSVs and COVID-19 cases in Taipei (lag –3), New Taipei (lag –2), Taoyuan (lag –2), Tainan (lag –1), Taichung (lag 0), and Kaohsiung (lag 0).	COVID-19	Google Trends
6	Eysenbach [40]	Found a strong correlation (Pearson *r*=0.91) between the number of clicks on a keyword-triggered link in Google with epidemiological data from Canada’s flu season of 2004-2005.	Influenza	Google Trends
7	Yang et al [51]	To improve the existing Google Flu Trends (GFT), they proposed an influenza tracking model, ARGO (AutoRegression with Google search data), that uses publicly available online search data. Besides having a rigorous statistical foundation, ARGO outperforms the latest GFT version. Not only does ARGO incorporate seasonality in influenza epidemics but it also captures changes in online search behavior over time.	Influenza	ARGO
8	Cook et al [52]	Evaluated the accuracy of each US GFT model by comparing weekly estimates of influenza-like illness (ILI) activity with the US Outpatient Influenza-like Illness Surveillance Network (ILINet). They calculated the correlation and RMSE between model estimates and ILINet for four seasons: pre-H1N1, Summer H1N1, Winter H1N1, and H1N1 overall. Both models’ estimates were highly correlated with ILINet pre-H1N1 and over the entire surveillance period, although the original model underestimated the magnitude of ILI activity during the pre-H1N1 phase. The updated model was more correlated with ILINet than the original model during Summer H1N1 (*r *= 0.95 and 0.29, respectively).	Influenza	Google Trends
9	Yuan et al [53]	Used Baidu, a popular Chinese search index, to model and monitor influenza activity in China. A comprehensive technique was presented for (1) keyword selection, (2) keyword filtering, (3) index composition, and (4) modeling and detection of influenza activity in China. Sequential time series for the selected composite keyword index was significantly correlated with official Chinese influenza cases. Further, 1-month-ahead prediction of flu cases had a considerably small prediction error (mean absolute percent error<11%).	Influenza	Baidu search index
10	Dong et al [36]	Used DXY, an online platform of the Chinese medical community, as a primary data source to develop an online interactive dashboard. The dashboard is hosted by the Center for Systems Science and Engineering (CSSE) at Johns Hopkins University, United States. They monitored various Twitter feeds, online news services, and direct communication sent through the dashboard to identify new cases.	COVID-19	DXY, Twitter feeds, online news services
11	Signorini et al [44]	To explore public concerns regarding rapidly evolving H1N1 activity, used Twitter data and support vector regression to show that estimates of ILI accurately tracked reported disease levels. They retrieved a large sample of public tweets that matched a set of flu-related search terms.	Influenza	Twitter
12	Chew and Eyesenbach [54]	Proposed and evaluated a complementary infoveillance approach using Twitter during the 2009 H1N1 pandemic. They performed a content analysis of tweets and validated Twitter as a real-time content and sentiment tracking tool. Infovigil, an infoveillance technology, was used to record more than 2 million Twitter postings with the terms “swine flu” and “H1N1.” According to content analysis, resource-related posts were most commonly shared (52.6%). Misinformation occurred in 4.5% of the cases. News websites were the most popular sources (23.2%), while government and health agencies were linked only 1.5% of the time.	Influenza	Twitter
13	Zhou et al [35]	Used logistic regression models to explore the risk factors associated with in-hospital deaths. They utilized a retrospective, multicenter cohort study that included all adult inpatients with laboratory-confirmed COVID-19.	COVID-19	Electronic health (medical) records
14	Huang et al [39]	This descriptive study detailed descriptive statistics of clinical features of patients infected with COVD-19 in China as extracted from electronic medical records.	COVID-19	Electronic health (medical) records
15	Williamson et al [49]	Developed OpenSAFELY, a secure health analytics platform that maintains patient data in the current data center of a significant provider of primary care electronic health records and serves 40% of all patients in England. OpenSAFELY was used to examine factors associated with COVID-19–related deaths; 10,926 COVID-19–related deaths were pseudonymously linked to primary care records of 17,278,392 persons.	COVID-19	Electronic health (medical) records
16	Wu et al [50]	Used data on monthly airline bookings from the Official Aviation Guide and data on human mobility across more than 300 prefecture-level Chinese cities from the Tencent database. The reports released by the Chinese Center for Disease Control and Prevention provided information on confirmed cases. A susceptible-exposed-infectious-recovered (SEIR) model was used to simulate the epidemics in China’s main cities. They concluded that, with a lag time of roughly 1-2 weeks behind the Wuhan outbreak, epidemics were already expanding exponentially in several large cities throughout China, assuming the transmissibility of SARS-CoV-2 was identical domestically and over time.	COVID-19	Tencent Migration data
17	Santillana et al [43]	Presented an ensemble-based machine learning method that leverages data from various sources, including Google searches, Twitter microblogs, and near real-time hospital visit records, to provide nowcast and forecast estimates of influenza activity in the United States. Their method combines multiple ILI activity estimates, generated independently with each data source, into a single prediction of ILI. Evaluation of the predictive ability of their method suggests that it outperforms every prediction using each data source independently. Additionally, it generated estimates 2 and 3 weeks ahead of time with comparable accuracy to real-time forecasts from an autoregressive model and predictions 1 week ahead of GFT’s real-time estimates.	Influenza	Google searches, Twitter microblogs, and near real-time hospital visit records
18	Li et al [42]	Evaluated the predictive value of search data from Google Trends and two Chinese social media platforms, Weibo index and Baidu index, for the COVID-19 epidemic in China. They observed that the peak of internet searches and social media data about the COVID-19 outbreak occurred 10-14 days earlier than the peak of daily incidences in China. Internet searches and social media data were highly correlated (*r*>0.89) with daily incidences.	COVID-19	Google Trends, Weibo index, Baidu index
19	Cervellin et al [56]	Compared the reliability of Google Trends in different clinical settings for common diseases with lower media coverage and for less common diseases attracting major media coverage. They carried out a Google Trends search using the keywords “renal colic,” “epistaxis,” and “mushroom poisoning.” Additionally, a second search was carried out for three clinical conditions (ie, “meningitis,” “*Legionella pneumophila* pneumonia,” and “Ebola fever”). No correlation was observed between Google Trends and epidemiology of renal colics, epistaxis, and mushroom poisoning. When searching the term “mushroom” alone, the Google Trends search generated a seasonal pattern, almost overlapping with the epidemiological profile.	Meningitis, *Legionella* pneumonia, and Ebola	Google Trends
20	Teng et al [55]	Developed a dynamic forecasting model for Zika virus (ZIKV) based on Google Trends. A strong correlation was found between Zika-related Google Trends and the cumulative numbers of reported cases (confirmed, suspected, and total cases *P*<.001). Further, an autoregressive integrated moving average (ARIMA) model (0,1,3) was fitted for the dynamic estimation of ZIKV outbreaks. The forecasting results indicated that the ARIMA model, which used the online search data as the external regressor to enhance the forecasting model, is quite similar to the actual data during the Zika virus epidemic.	Zika	Google Trends

## Discussion

### Principal Findings

Novel big data streams have created interesting opportunities for infectious disease monitoring and control. The review of the top 20 papers suggests the domination of high-volume electronic health records and digital traces such as internet searches and social media. Of note is the relatively increased use of Google Trends. Most studies used Google Trends data by correlating them with official data on disease occurrence, spread, and outbreaks. Some of these studies further adopted nowcasting for disease surveillance. However, using Google Trends for forecasts and predictions in infectious diseases epidemiology fills a gap in the extant literature. Few studies have gone as far as predicting incidents and occurrences, even though data on reported cases of various health concerns and the associated Google Trends data have been correlated in many studies. Predicting the future is hard; hence, more reliable and efficient methodologies are needed for forecasting infectious disease outbreaks.

There are a few drawbacks to digital trace data that should be considered. Many of these data streams miss demographic information such as age and gender, which is essential in almost any epidemiological study. Besides, they represent a growing but still limited population segment, with infants unfeatured and fewer older adults than younger people. Geographic heterogeneity in coverage exists, with underrepresentation in developing countries, although these biases tend to fade and are arguably less pronounced than those found in traditional global surveillance systems. Further, the retrieved data are subject to spatial and temporal uncertainty. Accordingly, hybrid systems that supplement rather than replace conventional surveillance systems as well as improve prospects for accurate infectious disease models and forecasts should be developed.

Most studies, except for those in the United States and China, were conducted in the European context. Thus, more studies need to test the utility of these big data streams for infectious disease epidemiology in the context of more countries, especially in Africa. Future research questions should ask if any cross-cultural differences between countries affect the adoption and use of big data in infectious disease epidemiology.

The vast majority of infectious diseases have a global distribution. Apart from the coronavirus, influenza, Zika, and Ebola virus outbreaks that are featured in our review, the utility of these big data sources for more infectious diseases should be studied.

### Limitations

A few limitations were inherent in our study. First, like any bibliometric study, we are limited by the search terms and database used. This study utilized English publications from the WoS core collection; therefore, relevant publications may have been missed. However, our choice of WoS was informed by its greater coverage of high-impact journals. Second, some studies may have been published after we concluded document extraction. Accordingly, this study does not claim to be exhaustive but rather extensive.

### Future Research Agenda and Conclusions

The bibliometric study identified the United States and China as research leaders in this field, with most affiliations from the Harvard Medical School and the University of California. Top authors were Zhang Yi and Li Xingwang. *Journal of Medical Internet Research* and *PLoS One* are the most productive and influential journals in this field. Internet searches and social media data are the most utilized data sources. COVID-19 and influenza were the most studied infectious diseases. The main research themes in this area of research were disease monitoring and surveillance, utility of electronic health (or medical) records, methodology framework for infodemiology tools, and machine/deep learning. Most research papers on big data in infectious diseases epidemiology were published in outlets related to computer science, public health, and health care services.

Opportunities for future research are revealed directly from the results of this study. Integrating multiple surveillance platforms, including big data tools, are critical to better understanding pathogen spread. It is also paramount for the research needs to align with a global view of disease risk. The risk of infectious disease is globally shared in an increasingly connected world. The COVID-19 pandemic, including the rapid global circulation of evolved strains, has emphasized the need for an interdisciplinary, collaborative, global framework for infectious disease research and control. There is a need to empower epidemiologists and public health scientists to leverage insights from big data for infectious disease prevention and control.

## Abbreviations

WoS: Web of Science
